# Response of the Human Circadian System to Millisecond Flashes of Light

**DOI:** 10.1371/journal.pone.0022078

**Published:** 2011-07-08

**Authors:** Jamie M. Zeitzer, Norman F. Ruby, Ryan A. Fisicaro, H. Craig Heller

**Affiliations:** 1 Department of Psychiatry and Behavioral Sciences, Stanford University, Stanford, California, United States of America; 2 Mental Illness Research, Education and Clinical Center, VA Palo Alto Health Care System, Palo Alto, California, United States of America; 3 Department of Biology, Stanford University, Stanford, California, United States of America; 4 School of Humanities and Sciences, Stanford University, Stanford, California, United States of America; Vanderbilt University, United States of America

## Abstract

Ocular light sensitivity is the primary mechanism by which the central circadian clock, located in the suprachiasmatic nucleus (SCN), remains synchronized with the external geophysical day. This process is dependent on both the intensity and timing of the light exposure. Little is known about the impact of the duration of light exposure on the synchronization process in humans. *In vitro* and behavioral data, however, indicate the circadian clock in rodents can respond to sequences of millisecond light flashes. In a cross-over design, we tested the capacity of humans (n = 7) to respond to a sequence of 60 2-msec pulses of moderately bright light (473 lux) given over an hour during the night. Compared to a control dark exposure, after which there was a 3.5±7.3 min circadian phase delay, the millisecond light flashes delayed the circadian clock by 45±13 min (p<0.01). These light flashes also concomitantly increased subjective and objective alertness while suppressing delta and sigma activity (p<0.05) in the electroencephalogram (EEG). Our data indicate that phase shifting of the human circadian clock and immediate alerting effects can be observed in response to brief flashes of light. These data are consistent with the hypothesis that the circadian system can temporally integrate extraordinarily brief light exposures.

## Introduction

Exposure to bright light at night has multiple effects on the human hypothalamus, including phase shifting circadian rhythms, altering hormone production, and enhancing alertness [1]. The effects of light are dependent on both the intensity [2], [3] and timing [4] of light exposure. Transduction of light signals from the retina to the central circadian clock, located in the hypothalamic suprachiasmatic nucleus (SCN), is mediated through a network of retinal cones, rods, and melanopsin-expressing intrinsically photosensitive retinal ganglion cells (ipRGC) [5]. This network also projects to other hypothalamic regions [5]. To examine light-induced changes in human hypothalamic function, typical protocols use hours or, occasionally, minutes of bright light exposure [1]. Behavioral data from nocturnal rodents [6]–[8] suggest, however, that the human hypothalamus might be able to respond to light that is thousands of times shorter. As such, we examined the capacity of the human hypothalamus to respond to millisecond flashes of light.

## Methods

### Ethics Statement

This study and all related procedures described herein were reviewed and approved by the Stanford University Institutional Review Board and conform to the principles expressed in the Declaration of Helsinki. Subjects signed informed consent forms prior to any procedures.

Seven healthy adults (aged 18–48 years; 6 male, 1 female) participated in a pair of two-day in-laboratory sessions that were separated by at least two weeks. Subjects had no active disease processes; they were non-smokers and had normal hearing. Subjects did not have sleep disorders (Pittsburgh Sleep Quality Index score ≤5 [9]) nor did they routinely take medication that could impact their sleep, including daily use of antihistamines or antidepressants. Subjects were of intermediate chronotype as determined by the Horne-Östberg questionnaire [10].

For the two weeks prior to coming into the laboratory, subjects were required to maintain a regular, at-home sleep schedule. Regularity was defined as keeping sleep and wake times that were 7–9 hours apart and each of which did not differ from a predetermined target time by more than 30 minutes. Subjects completed a daily log of sleep and wake times and wore a wrist actigraph (Actiwatch2, Philips-Respironics, Bend OR) during this two-week at-home portion of the protocol. The actigraph records three-dimensional arm movement; such data are useful for approximating sleep and wake [11]. These actigraph units were also equipped with an ambient illumination detector. At entry into the laboratory, actigraph and illuminance data were analyzed and used to confirm the self-reported sleep log data. All subjects maintained the required at-home sleep schedule. Subjects did not take non-steroidal anti-inflammatory medications on the day prior to or during their in-laboratory stay as these might affect plasma melatonin concentrations [12]. This rigorous at-home sleep schedule acts to stabilize the relationship between the circadian clock and the timing of sleep and wake. In individuals who undertake such a schedule and who are not an extreme chronotype, there is a highly predictable phase angle relationship between the onset of sleep and the timing of the circadian clock [13]. Additionally, the regularization of light exposure that occurs with schedule maintenance will maximize the oscillation amplitude of the circadian clock [14]. The average bed and wake times were calculated and the midpoint between these two times was used as the midpoint of an eight hour episode of darkness while in the laboratory. The one female subject entered the laboratory within the first three days after menses onset.

Subjects spent their in-laboratory portion of the study in a time isolation suite at the VA Palo Alto Health Care System. The room has an en suite bathroom and all of the lighting is controlled by a technician located outside of the room. The walls of the room are coated with a highly reflective white, titanium dioxide-based paint. There are no time cues within the suite (*e.g.*, windows, clocks, radio, internet, television). Room temperature was maintained within a normal ambient range ±3°C. Corneal illuminance was no greater than 10 lux during scheduled wake (except experimental light exposure, see below) and was dark (<0.03 lux) during scheduled sleep.

Subjects came to the laboratory seven hours after their habitual wake time (relative clock times herein are normalized to a wake time of 08:00). Starting two hours after arrival and continuing until habitual bedtime (17:00–24:00), subjects engaged in a constant posture protocol in dim light. During the constant posture, subjects remained in bed with the head of the bed slightly elevated (30°±10° per subject comfort, but constant throughout once set within the first hour of the constant posture). If the subject needed to urinate or defecate, a urinal or bed pan was provided as appropriate. Instead of receiving a dinner, subjects received hourly isocaloric snacks and isovolumetric water rations that were calculated [15] as replacements for the evening meal. The use of the constant posture protocol helps to minimize or exclude behaviors (*e.g.*, posture) that might influence melatonin concentrations found in saliva [16].

**Figure 1:**
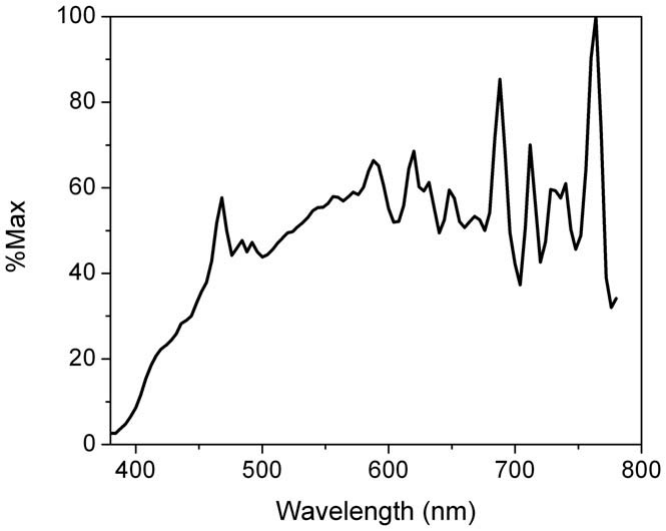
Spectral distribution of tungsten lamp as measured by a PR-650 SpectraScan Colorimeter (Photo Research, Chatsworth CA). Data were normalized to maximum power and plotted over the visible spectrum.

During the constant posture, saliva was collected every 30 minutes (Salivettes; Sarstedt, Nümbrecht Germany) and vigilance was monitored hourly using the Stanford Sleepiness Scale (SSS) [17] and a 10-minute audio version of the Psychomotor Vigilance Test [18], [19]. Prior to habitual bedtime, subjects were fitted with electrodes to record the polysomnogram (C3, C4, O1, O2, A1, A2, electrocardiogram, bilateral electro-oculogram, chin electromyogram; Siesta, Compumedics, Charlotte NC). At habitual bedtime, subjects were asked to try to sleep, maintaining their same posture. One hour and fifty minutes after lights-out, subjects were awoken into a dark room (01:50). They completed an SSS then an aPVT; technicians interacted with the subjects while wearing infrared sensitive goggles with infrared emitter. For the next hour (02:00–03:00), subjects were subjected to one of two experimental protocols: (1) continuous darkness or (2) 60 2-msec pulses of 473 lux light with each pulse being spaced by 60 seconds of darkness. Each subject experienced both protocols, but on separate visits, with the order randomized. Light flashes were produced by a tungsten lamp with a broad emission spectrum (Figure 1). The lamp was controlled by a custom unit designed and built by Dr. Howard Davidson. Illuminance was measured with a Spectra Professional IV-A photometer (Spectra Cine, Burbank CA) and confirmed with an ILT1700 Research Radiometer/Photometer (International Light Technologies, Peabody MA). During the last 10 minutes of the experimental stimulus, subjects took an aPVT followed by an SSS. Subjects provided a saliva sample in the dark before the onset of the experimental stimulus and a second sample at the end of the experimental stimulus, after which they were allowed to sleep in the dark for the next six hours. Subjects were awoken into dim (<10 lux) illuminance and were ambulatory for nine hours (08:00–17:00), during which time they received a standard hospital breakfast and lunch. From nine hours after waketime until two hours after habitual bedtime (17:00–02:00), subjects engaged in a second constant posture protocol during which they provided half-hourly saliva samples and had their alertness assessed (SSS, aPVT) hourly. Subjects were then discharged.

### Assessment of circadian phase

Saliva samples were immediately frozen and stored at −80°C. Samples were later unfrozen, spun in a centrifuge, and the filtered saliva decanted. Salivary melatonin concentrations were determined using a commercially available radioimmunoassay (ALPCO, Salem NH). Inter- and intra-assay coefficients of variation were 9.8% and 7.9% respectively. Onset (phase) of the nocturnal melatonin surge was calculated as the time at which salivary melatonin concentrations surpassed a subject-specific threshold, calculated as the average of the first three daytime concentrations plus twice the standard deviation of these values [20]. Change in phase was calculated as the difference between melatonin onset on day 1 and day 2, making delays in phase negative. Melatonin suppression was calculated as the difference in melatonin concentration in the saliva sample obtained at the end of and immediately before the experimental light stimulus, divided by the melatonin concentration in the saliva sample obtained immediately before the experimental light stimulus.

### Assessment of alertness

The SSS is a seven-point Likert-like scale that examines proximal feelings of sleepiness. The aPVT is a serial reaction time test that measures sustained alertness. aPVT data were analyzed using the manufacturer software (React, Ambulatory Monitoring, Ardsley NY) from which the following variables were derived: average and median reaction time, number of lapses (non-response for 500 msec), duration of responses in the lapse domain (mean inverse of reaction times from slowest 10% of trial), shifts in optimum reaction time (mean inverse of reaction times from fastest 10% of trial), and time-on-task decrement (slope of linear regression fit to inverse reaction time). Objective changes in brain activity were also quantitated using electroencephalography and polysomnography. Nocturnal electrophysiology data were scored for sleep staging using standard methodology [21] by a single expert technician. EEG data during the 10-minute aPVTs that immediately proceeded and occurred at the end of the experimental stimulus were also examined for frequency-specific changes (PRANA, PhiTools, Chicago IL). EEG data derived from each cortical electrode (C3/4, O1/2) was referenced to an electrically neutral auricular electrode. Data were transformed using a fast Fourier transform (FFT) with a window length of 2 s, overlap of 0%, using a Hanning window type with a maximum frequency of 50 Hz, followed by Welch averaging with a mean averaging type on 30 s for a threshold of 50%, and feature extraction on both power (absolute IU^2^ and relative %) and frequency (mean Hz and peak Hz). Using this method for each 30 s bin, the absolute power in the different frequency spectra was calculated. Frequency spectra were grouped, as is typical in such analyses, into frequency bands: delta (0.5–4 Hz), theta (4–7.5 Hz), alpha (8–12.5 Hz), sigma (12–14 Hz), beta (14–29 Hz) and gamma (30–40 Hz). EEG data with artifact were removed from the analyses; only data from artifact-free periods of wake were examined.

All data are presented as mean ± SD. Specific statistical tests are noted within the text.

## Results

We were able to detect onset of melatonin in six of seven subjects on both visits; in one subject on one visit, melatonin onset did not occur until, presumably, after sampling ceased. This subject was excluded from all further analyses. In the remaining six subjects, there was no difference in the interval (phase angle) between the onset of melatonin and the beginning of the two experimental light stimulation protocols (5 h 9 min±50 min vs. 4 h 59 min±65 min, flash vs. dark; p = 0.64, paired *t*-test), indicating that the two experimental light exposures were given at the same circadian phase (in the delay region of the phase response curve) in all subjects. Given the phase-dependent effects of light on circadian timing [4], this is a critical consideration in the comparability of the two light stimulation protocols.

**Figure 2:**
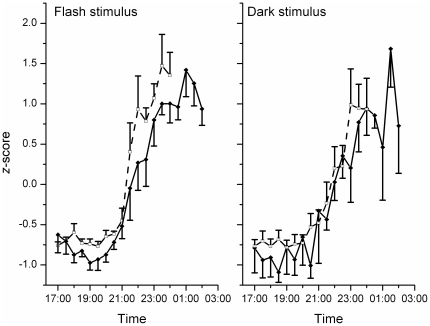
Phase shifts following the flash or dark stimuli. Average salivary melatonin profiles pre- (dashed lines) and post- (solid lines) exposure to either a series of flashes (left panel, 60 2-ms flashes, 1 per minute) or continuous darkness (right panel) for an hour are shown. Data were *z*-score transformed prior to averaging and aligned to a habitual bedtime of 24:00.

In the flash protocol, subjects experienced a −45±13 min phase change (*i.e.*, delay) whereas in the darkness condition, subjects experienced a −3.5±7.3 min phase change (p<0.01, paired *t-*test) (Figure 2). This sequence of flashes was unable to suppress melatonin secretion as there was no difference in salivary melatonin concentrations following the flash and dark conditions (reductions of 34±40% and 8.7±43%, respectively, p = 0.54, paired *t-*test; n = 5 due to assay failure), despite robust suppression of melatonin in ∼500 lux of continuous light [2].

**Figure 3:**
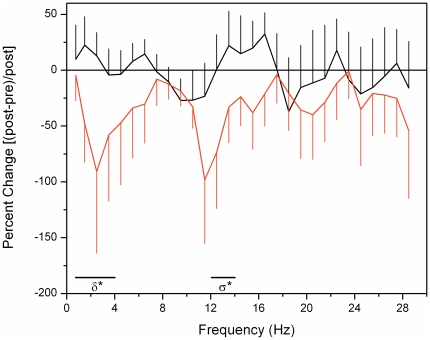
Change in EEG power in response to flash or dark stimuli. Percentage change in EEG power from before to after the dark (black) or flash (red) stimuli are presented. Significant (*p<0.05) differences between conditions are found in the delta (δ) and sigma (σ) bands.

There was a general decrease in EEG power across all frequencies (0.5–30 Hz) (Figure 3) after exposure to the flash protocol as compared to the dark protocol (p<0.001, two-way repeated measure ANOVA). *Post-hoc* comparison of individual frequency bands indicated that this phenomenon was significant in the delta (0.5–4 Hz) and sigma (12–14 Hz) frequency bands (p's<0.05, paired *t*-tests). In comparing the relative changes in the delta and sigma frequency bands with the changes in subjective (SSS) and objective (aPVT) alertness measures, changes in sigma were not correlated with changes in either SSS or aPVT measures (p's>0.44, |r|'s<0.24; Spearman Rank correlation). Changes in the delta frequency were also not correlated with SSS changes (p = 0.90, r = −0.04), but were well correlated with changes in mean (r = 0.58) and median (r = 0.60) reaction times on the aPVT (p's<0.05) such that reaction times were faster when there was less EEG delta activity after the flash stimulus.

## Discussion

Following exposure to a sequence of 2-msec flashes of moderately bright (473 lux) light occurring once per minute for 60 minutes (*i.e.*, 0.12 s of light over an hour) during the biological night, we observed a significant phase delay in the timing of the circadian pacemaker. It is possible that the extent of the phase change would be different if multiple circadian cycles following the stimulus were monitored, though this is not typical in human experiments nor do we expect transients in phase delays, though this is a possibility with such a novel stimulus. Unusual transients were not specifically reported in previous studies of the effects of light flashes in rodents [7], [8], [22]. Changes in EEG delta and sigma power were also observed following the flash sequence, with decreases in delta power being associated with improved subjective and objective measures of alertness. These data demonstrate that the human hypothalamus has the capacity to respond to a sequence of millisecond flashes of light.

While not directly compared in this study, others have observed previously that one hour of continuous bright (10,000 lux) light given during the early biological night (similar stimulus timing as in the current study) produces an 82 minute delay in circadian phase [23]. That light was considerably brighter (10,000 lux vs. 473 lux) and was continuous versus a sequence of flashes (3600 s vs. 0.12 s); nevertheless, the flash protocol used in this study resulted in 45% of the phase shift previously determined for continuous bright light. Thus, with light 30,000 times briefer and more than 20 times dimmer, we were able to obtain almost half the change in phase. In a separate study, we found that administration of 6.5 hours of light of the intensity examined here would have generated a 154 minute delay in circadian phase [2]. Thus, with light exposure 195,000 times briefer, we were able to obtain 29% of the change in phase.

Data from mice [6], rats [7], and hamsters [8] all indicate that the circadian clocks of non-human mammals have the capacity to respond to a sequence of millisecond flashes. Notably, in mammals, a single millisecond flash does not significantly change circadian phase [6], [24]. This indicates that a sequence of light flashes is “seen” by the circadian clock as more of a continuous stimulation than a series of discrete stimulations. This process can be referred to as temporal integration. Electrophysiologic data from ipRGC [25]–[27] and SCN neurons [28], [29] indicate that either or both of these loci might have the capacity to integrate discrete light stimulation as both the ipRGC and SCN neurons display prolonged excitation following cessation of stimulation. Our data do not discriminate where such temporal integration is occurring, but they do impute that such integration occurs in humans. Further experiments will be required to examine the range and extent of temporal integration of light by the human circadian system.

Previous experiments in hamsters indicate that behavioral changes in sleep/wake activity can be elicited by a sequence of brief flashes of light [30]. In this experiment, we observe improvements in subjective and objective alertness and changes in the EEG that are consistent with increased alertness. In response to the sequence of light flashes, there was a decrease in power in both sigma and delta activity during wake. The changes in sigma power appeared to be unrelated to changes in our measures of subjective and objective alertness, but the decrease in delta power was well correlated with improvements in alertness. Delta power is theorized to represent “sleep pressure” [31] so the correlation between decreased delta power and increased alertness is not altogether unexpected. These data suggest that even brief exposure to light at night has a significant effect on alertness.

We did not observe a significant effect of the flash sequence on acute suppression of salivary melatonin concentrations. Previous data indicate that exposure to continuous light, the intensity of which was similar to that used here, can suppress melatonin secretion [2] and that such suppression occurs quite rapidly following light exposure [32]. Given the high degree of variability, a *post-hoc* power calculation determined that we could have observed a difference of 60% between the flash and dark exposures. Thus, there may have been a difference in melatonin suppression between the two conditions that was below our ability to discriminate. The pathway that underlies the acute effects of light on release of pineal melatonin involves the hypothalamus, spinal cord, and superior cervical ganglion. If temporal integration of photic information occurs in the SCN, but not in the ipRGC, and if the hypothalamic circuitry involved in melatonin suppression does not involve the SCN, then it is possible that there is a genuine lack of melatonin suppression in response to this flash sequence. Recent electrophysiologic evidence supports the idea that there may be distinct patterns of light responsivity in the SCN and other hypothalamic nuclei [33], which could help to explain our observed dichotomy between melatonin suppression and phase shifting. Future experiments with larger numbers of subjects and different flash durations will be necessary to discriminate these possibilities.

Our data indicate that, quite distinct from the mechanisms underlying conscious visual perception, human circadian and alerting responses to light appear to be able to be integrated over time. These data represent a novel mechanism by which therapeutic intervention by light (*e.g.*, for shift work sleep disorder, delayed sleep phase syndrome) could be exploited, especially under circumstances in which exposure to continuous bright light is either inefficient or untenable.
